# Respiratory Syncytial Virus Epidemiology in Transition: Five Years of Profound Change

**DOI:** 10.3390/v18030360

**Published:** 2026-03-16

**Authors:** Emanuele Castagno, Irene Raffaldi

**Affiliations:** Department of Pediatric Emergency, Regina Margherita Children’s Hospital, 10126 Turin, Italy; iraffaldi@cittadellasalute.to.it

The landscape of respiratory syncytial virus (RSV) epidemiology has undergone unprecedented transformation over the past five years, fundamentally altering our understanding of viral transmission dynamics, disease burden across age groups, and preventive strategies. The second edition of this Special Issue on RSV epidemiological surveillance arrives at a pivotal moment, as the global health community grapples with the enduring consequences of pandemic-era disruptions whilst simultaneously witnessing the real-world implementation of novel prophylactic interventions. The contributions assembled herein provide critical insights into these evolving dynamics, offering evidence-based perspectives that will inform public health policy and clinical practice for years to come.

The COVID-19 pandemic precipitated striking perturbations in RSV circulation patterns that have reverberated through all age demographics. Following initial suppression during 2020, an interseasonal surge occurred in 2021, predominantly affecting children and young adults after relaxation of non-pharmaceutical interventions [1]. Notably, this resurgence showed age-specific heterogeneity, with older adults experiencing disproportionately lower infection rates—likely attributable to sustained adherence to protective behaviours within this vulnerable population [1]. These observations underscore the complex interplay between population immunity, behavioural factors, and viral transmission dynamics.

In children, the disruption of typical seasonal patterns has resulted in cohorts of infants and young children with reduced prior RSV exposure, creating what some have termed an ‘immunity debt’. This phenomenon has manifested as larger-than-expected case numbers in subsequent seasons, placing considerable strain on paediatric healthcare systems globally. Conversely, adult and elderly populations have demonstrated more variable patterns, with some regions reporting delayed or attenuated RSV seasons, whilst others have observed intensified activity. The establishment of robust surveillance platforms, such as population-based record linkage systems, has proven essential for capturing these nuanced epidemiological shifts and identifying high-risk subgroups across the age spectrum [2].

The past five years have illuminated the intricate relationships between RSV and other respiratory pathogens, particularly influenza and SARS-CoV-2. During periods of stringent COVID-19 mitigation measures, near-complete suppression of RSV and influenza transmission was observed globally, providing compelling evidence for viral interference mechanisms and the efficacy of non-pharmaceutical interventions against respiratory viruses broadly. However, the subsequent return to endemic circulation has revealed complex co-circulation patterns that challenge traditional assumptions about viral competition.

Emerging evidence suggests that the timing and intensity of RSV seasons may be influenced by preceding or concurrent circulation of other respiratory viruses, with implications for healthcare capacity planning and vaccine deployment strategies. The phenomenon of ‘immune imprinting’ from early-life viral exposures may also contribute to altered susceptibility patterns in populations experiencing disrupted exposure histories. Such observations necessitate integrated surveillance approaches that monitor multiple respiratory pathogens simultaneously, enabling detection of epidemiological interactions that single-pathogen surveillance systems might overlook.

The introduction of nirsevimab represents a watershed moment in RSV prevention, with accumulating real-world evidence demonstrating substantial reductions in infant hospitalisations across diverse healthcare settings. Population-based studies from Spain have documented the impact of universal prophylaxis programmes across two RSV seasons, revealing significant decreases in both hospital admissions and primary care presentations [3]. Systematic reviews and meta-analyses of real-world effectiveness data consistently report robust protection against RSV-related lower respiratory tract infections, with effect sizes comparable to those observed in clinical trials [4].

Importantly, implementation studies have revealed critical insights regarding optimal deployment strategies, including the comparative effectiveness of catch-up versus at-birth immunisation schedules and the population-level impact of achieving high coverage rates [5]. These findings have profound implications for health economic evaluations and programme design, particularly in resource-constrained settings where targeted versus universal approaches must be carefully considered. The durability of protection throughout the RSV season and the potential for indirect effects through reduced transmission remain active areas of investigation that will inform future policy recommendations.

The papers in this Special Issue collectively advance our understanding of RSV epidemiology during a period of remarkable change. As we transition from pandemic disruption to a new equilibrium characterised by widespread availability of effective prophylaxis, continued vigilance through robust surveillance systems remains paramount. Future research must address persistent knowledge gaps, including long-term population-level effects of nirsevimab implementation, optimal strategies for adult and elderly populations, and the potential for viral evolution under selective pressure. The global RSV research community stands poised to translate these epidemiological insights into tangible improvements in disease prevention and clinical outcomes across all age groups.

## Data Availability

Not applicable.
